# Penalized-Likelihood PET Image Reconstruction Using Similarity-Driven Median Regularization

**DOI:** 10.3390/tomography8010013

**Published:** 2022-01-06

**Authors:** Xue Ren, Ji Eun Jung, Wen Zhu, Soo-Jin Lee

**Affiliations:** 1 Department of Electronic Engineering, Pai Chai University, Daejeon 35345, Korea; xueren@pcu.ac.kr (X.R.); 2023601@pcu.ac.kr (W.Z.); 2 Image Processing Group, Genoray, Company, Ltd., Seongnam 13230, Gyeonggi-Do, Korea; jejung@genoray.com

**Keywords:** image reconstruction, median regularization, non-local regularization, penalized likelihood, super-resolution reconstruction, positron emission tomography

## Abstract

In this paper, we present a new regularized image reconstruction method for positron emission tomography (PET), where an adaptive weighted median regularizer is used in the context of a penalized-likelihood framework. The motivation of our work is to overcome the limitation of the conventional median regularizer, which has proven useful for tomographic reconstruction but suffers from the negative effect of removing fine details in the underlying image when the edges occupy less than half of the window elements. The crux of our method is inspired by the well-known non-local means denoising approach, which exploits the measure of similarity between the image patches for weighted smoothing. However, our method is different from the non-local means denoising approach in that the similarity measure between the patches is used for the median weights rather than for the smoothing weights. As the median weights, in this case, are spatially variant, they provide adaptive median regularization achieving high-quality reconstructions. The experimental results indicate that our similarity-driven median regularization method not only improves the reconstruction accuracy, but also has great potential for super-resolution reconstruction for PET.

## 1. Introduction

Positron emission tomography (PET) has played an important role in molecular imaging by providing functional information about physiological process in the human body. The PET scanner detects gamma-ray photons created during the emission of positrons due to the breakdown of the radiotracer introduced in the patient body [1,2]. Then, the distribution of the radiotracer is visualized as a two-dimensional (2D) or 3D image by solving the inverse problem of image reconstruction from projectional views acquired at many different angles relative to the patient.

In clinical practice, since only a small amount of radiotracer must be injected into the human body to minimize the risk of radiation exposure, the quality of PET images is extremely poor compared to that of other imaging modalities, such as X-ray computed tomography (CT), whose radiation source is placed outside of the body. Moreover, in order to maintain an acceptable level of signal-to-noise ratio of the reconstructed image, the size of each detector unit must be designed to be large enough to collect a sufficient number of photons, which eventually limits the image resolution. Statistical noise due to scattered-coincidence and random-coincidence events, as well as several physical effects, also limits the spatial resolution in PET. Therefore, the use of a direct inversion method, such as the well-known filtered back-projection algorithm, which oversimplifies the PET reconstruction problem by ignoring the measurement noise and modeling the image formation process as line integrals through the radiotracer distribution, often leads to unacceptable noise amplification in the reconstructed image [2].

Over the last decades, a variety of reconstruction methods, which can improve the quality of reconstructed images by overcoming the limitation of the direct inversion method, have been developed. In particular, along with fast growing technologies in high-performance computing hardware, there has been increasing emphasis on developing model-based iterative reconstruction (MBIR) methods, which not only can incorporate a system model needed to account for physics of image formation, but also allow *a priori* information to be incorporated on the underlying source distribution. In fact, some of them are now limitedly used in clinical practice. For example, the Bayesian penalized likelihood algorithm (Q.Clear—General Electric Healthcare, Milwaukee, WI, USA) developed for General Electric PET scanners uses a point spread function model and a penalty function to reduce noise within each iteration. The overview of the development in MBIR methods for PET can be found in [3,4,5].

Recently, inspired by machine learning, example-based methods, such as dictionary learning-based methods [6,7] and convolutional neural network (CNN)-based methods [8,9], have been a topic in image denoising and have also attracted growing interests in medical imaging. While the example-based methods can be useful as post-processing tools for image denoising and artifact reduction in medical imaging, they have not yet been effectively incorporated into the tomographic reconstruction process, because they require large amounts of prior training pairs, some of which must be high-quality images, but are often unavailable in routine clinical practice. Moreover, it has been a challenging problem to incorporate deep neural networks into MBIR due to computational complexity in conjunction with the repeated time-consuming projection and back-projection operations. While deep learning methods with CNNs for tomographic reconstruction are being developed by many researchers and have also been applied to some MBIR methods, the claim that CNNs solve inverse problems as well as traditional MBIR methods do in medical imaging often remains unsubstantiated [10].

In this work, to improve MBIR for PET, we focus on the penalized-likelihood (PL) approach, where the penalty term (also known as the regularizer) takes the form of constraints on the local spatial structure of the underlying image. In general, regularizers are largely classified into two different types; local and non-local regularizers. While the local regularizers take the mean of a target pixel and its neighboring pixels, the non-local regularizers take the weighted mean whose weights reflect the “similarity” between the patch centered at the pixel being considered and its neighbor patch. Each type has its own advantages and disadvantages depending on its applications. For example, local regularizers are useful for preserving fine-scale edges when the penalty function takes a non-quadratic form, but local non-quadratic regularizers (LNQRs) [11,12] tend to produce spurious edges in monotonic areas when noise is present. On the other hand, non-local regularizers (NLRs) [13,14,15,16] are useful for preserving coarse-scale edges as well as monotonic areas but tend to oversmooth fine-scale edges [16].

In order to compromise the two different types of regularizers, one can consider linearly combining them using a control parameter. However, since the performance of each regularizer depends on the spatial characteristics of the underlying image, it is challenging to select an optimal value of the control parameter to balance between the two regularizers for all pixels.

In this work, we propose a different approach to PL-based regularization, which is expected to be more robust to noise than LNQRs while taking advantage of NLRs using patch similarities. Our proposed method first takes the median rather than the mean of pixels, which is known as median regularization. Our motivation for using the median regularizer lies in the fact that, unlike conventional LNQRs, where each pixel being updated is strongly affected by impulsive noise in its local neighbors, the median regularizer has a behavior whereby each pixel is attracted to its local median, thereby being less affected by such noise.

The idea of using the median regularizer for MBIR was first introduced in [17,18]. However, the methods proposed in [17,18] are based on a heuristic empirical approach rather than on a theoretical approach that solves an optimization problem. Therefore, the solutions obtained by these methods tend to diverge even for some reasonable values of the regularizing parameter. Later, Hsiao et al. [19] proposed a novel method of formulating a convex objective function for the PL-based reconstruction algorithm with a median regularizer and showed that an exact solution could be obtained by optimizing the objective function.

Here, we note that, while the median regularizer preserves edges as well as locally monotonic regions, it often results in a negative effect of removing fine-scale edges by eliminating any structure that occupies less than half of the median window elements. To overcome this problem, we extended the standard non-weighted median regularizer (NWMR) to the weighted median regularizer (WMR) [20,21,22,23], so that a pixel with a larger weight can be more probable to become a median than a pixel with a smaller weight.

In this work, inspired by the non-local means denoising approach [13], which exploits the self-similarities measured by the patch differences to reduce noise, the median weights for the WMR are determined by the patch differences within the neighborhood. However, unlike the NLR, instead of applying the patch differences to the smoothing weights, we applied them to the median weights. With this new approach, our PL-based reconstruction method was performed by similarity-driven median regularization.

To extend our application, we also considered super-resolution reconstruction where the resolution of PET images increases during the reconstruction process. This new application can be useful for increasing the spatial resolution of PET images without modification of PET scanner hardware. We show how super-resolution can be achieved within the PL framework and, finally, demonstrate its improved performance by using our proposed method.

## 2. Methods

### 2.1. Penalized-Likelihood Approach to PET Reconstruction

A dominant source of image degradation inherent in PET imaging is the noise due to the variation in the number of gamma rays emitted from a radiotracer. Since the total number of gamma photons emitted during a finite interval of time follows a Poisson distribution, the likelihood in the PL approach is expressed as a product of independent Poisson distributions, as follows:

(1) $$\mathrm{Pr}(g|f)=\prod_{i}\frac{{\bar{g}}_{i}^{g_{i}}e^{-{\bar{g}}_{i}}}{g_{i}!},$$

where f is the underlying source image, g is the observed projection measurements and $g_{i}$ is the *i*-th element of g representing the number of coincidence events detected by the *i*-th detector pair whose mean is denoted as ${\bar{g}}_{i}$. The image formation process, in this case, is expressed as ${\bar{g}}_{i}=\sum_{j}H_{ij}f_{j}+r_{i},$ where $H_{ij}\geq 0$ denotes the elements of the system matrix, each of which represents the probability of detecting an event originated at the *j*-th pixel by the *i*-th detector pair; $f_{j}$ is the *j*-th pixel of the underlying source distribution (or the underlying image); and $r_{i}$ is the mean number of background events such as scattered and random events.

By adding the regularizer R(f) to the likelihood in (1), the PL reconstruction method can be expressed as the following minimization problem:

(2) $$\hat{f}=\underset{f}{\arg\min}[-L(g|f)+\beta R(f)],$$

where L(g|f) is the log-likelihood (the log of Pr(g|f)) and β is a positive parameter (also known as a smoothing parameter) which controls the balance between the likelihood and the regularization terms.

### 2.2. Similarity-Driven Median Regularization

To formulate our idea of using the WMR, we followed the derivation of the PL algorithm with the “median prior” as proposed in [19], where the regularizer (or the prior) is modeled as a function that penalizes the differences between the pixels $f_{j}$ of the underlying object $f=\{f_{j};j=1,\ldots ,J\}$ and components $m_{j^{\prime }}$ of the auxiliary vector $m=\{m_{j^{\prime }};j^{\prime }\in N_{j}\}$ within the local neighborhood system $N_{j}$ of the *j*-th pixel, including itself. The auxiliary vector **m** is in register with **f** in such a way that $m_{j^{\prime }}$ interacts with $f_{j}$, so that $m_{j^{\prime }}$ is a neighbor of $f_{j}$ and vice versa. (See [19] for the details on the relationship between **f** and **m**.) Therefore, the WMR is expressed as

(3) $$R(f,m)=\sum_{j=1}^{J}\sum_{j^{\prime }\in N_{j}}w_{jj^{\prime }}\psi (f_{j}-m_{j^{\prime }}),$$

where $w_{jj^{\prime }}$ is the weight between j and $j^{\prime }$ and the penalty function is defined as $\psi \left(\xi \right)=\sqrt{\xi^{2}+\varepsilon }$, which is a differentiable approximation to the absolute function, $\underset{\varepsilon \to 0}{\lim}\psi \left(\xi \right)=|\xi |.$ Note that the penalty function in (3) is associated with penalizing the difference $f_{j}-m_{j^{\prime }}$ rather than $f_{j}-f_{j^{\prime }}.$ Note also that, if $w_{jj^{\prime }}$ is uniform over the entire image, (3) becomes the NWMR.

In this work, we propose a new WMR whose weights are determined by measuring the patch similarities within the neighborhood of the pixel to be updated. In this case, the new WMR becomes an adaptive version with space-variant weights which is different from the conventional WMR with fixed weights for all the pixels in the image. We call this regularizer the similarity-driven median regularizer (SDMR). Inspired by the non-local means approach [13], when the median window is centered at the pixel *j*, the median weights can be calculated as follows:

(4) $$w_{jj^{\prime }}=\frac{{\tilde{w}}_{jj^{\prime }}}{\sum_{j^{\prime }\in N_{j}}{\tilde{w}}_{jj^{\prime }}},\;\mathrm{where}\;{\tilde{w}}_{jj^{\prime }}=\exp\left(-\frac{{\left\|\rho (N_{j})-\rho (N_{j^{\prime }})\right\|}^{2}}{\delta^{2}}\right).$$

In (4), $N_{j}$ is the neighborhood system of the pixel *j*, $\rho (N_{j})$ is the patch centered at the pixel *j* and δ is a positive parameter. The difference between the two patches $\rho (N_{j})$ and $\rho (N_{j})$ centered at the pixels *j* and *j’*, respectively, is calculated by

(5) $$\Delta \rho_{jj^{\prime }}≜{\left\|\rho \left(N_{j}\right)-\rho \left(N_{j^{\prime }}\right)\right\|}^{2}=\sum_{p=1}^{P}{\left(f_{j(p)}-f_{j^{\prime }(p)}\right)}^{2},$$

where *P* is the total number of pixels in a patch and $f_{j(p)}$ and $f_{j^{\prime }(p)}$ are the *p*-th pixels in the patches $\rho (N_{j})$ and $\rho (N_{j^{\prime }})$, respectively.

**Figure 1:**
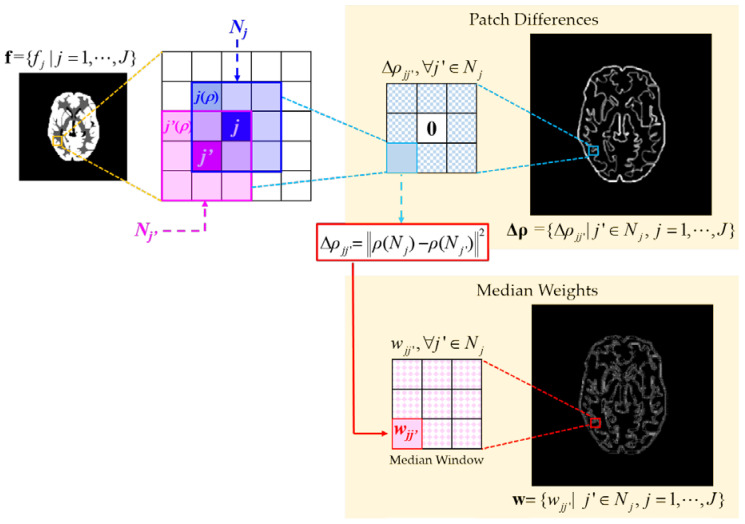
Illustration of similarity-driven median weights. Given the PET image f, the median weights w are determined by the patch similarities Δρ.

Figure 1 illustrates self-similarity driven median weights whose values for the pixel *j* are determined by the patch differences Δρ**,** which are measured by calculating the difference between the center patch $\rho (N_{j})$ and its neighbor patch $\rho (N_{j^{\prime }})$ for all $j^{\prime }\in N_{j}$. The median weights are calculated in such a way that the higher the relative similarity between the center patch and its neighbor patch is, the more probable the median of the neighbor patch (designated as $m_{j^{\prime }}$) is to be chosen as the median of the center patch within a median window. If the difference between the center patch $\rho (N_{j})$ and its neighbor patch $\rho (N_{j^{\prime }})$ is negligible, the value of ${\tilde{w}}_{jj^{\prime }}$ is close to 1, which is the largest possible value of ${\tilde{w}}_{jj^{\prime }}$ in the median window. In this case, a relatively larger weight is assigned to $m_{j^{\prime }}$, so that $m_{j^{\prime }}$ becomes more probable to be chosen as the median of $\rho (N_{j})$. In contrast, when the neighbor patch is dissimilar to the center patch, the value of ${\tilde{w}}_{jj^{\prime }}$ is close to 0. In this case, a relatively smaller weight is assigned to $m_{j^{\prime }}$, so that $m_{j^{\prime }}$ becomes less probable to be chosen as the median of $\rho (N_{j})$. This implies that, for the homogeneous regions consisting of nearly uniform patches, the SDMR behaves similar to the NWMR by using almost-uniform weights. On the other hand, for the non-homogeneous regions consisting of different patches, the SDMR preserves fine-scale edges more accurately than the NWMR by using the nonuniform weights whose values are close to one only when the similarities are very high.

In this work, we also applied the SDMR to the super-resolution (SR) reconstruction problem. Our motivation of considering SR is based on the recent development of multimodal medical imaging systems, such as PET combined with X-ray CT (PET/CT) and PET combined with magnetic resonance imaging (PET/MRI), where the image resolution of PET is much lower than that of CT or MRI. Most of the SR techniques used in PET reconstruction are based on the multi-frame SR (MFSR) technique that combines the multiple low-resolution (LR) images generated either by acquiring the projection data from different points of views or by shifting the reconstruction pixel grid during the image reconstruction process [24,25,26,27]. In contrast, we present a different approach to SR reconstruction for PET, where a high-resolution (HR) image is reconstructed from a single set of standard LR projections, which is similar to single-frame SR (SFSR) [28,29,30,31] as opposed to MFSR in non-tomographic SR applications. To increase the pixel resolution, upscaling is performed by back-projecting the projection measurements into the HR image space modeled on a finer grid [32].

### 2.3. Optimization of PL-SDMR Algorithm

For our PL reconstruction, the key to SR is to estimate the underlying HR image $f^{H}$ from the projections g acquired by LR detectors. In this case, the PL algorithm using the SDMR defined above reduces to a joint estimation of both $f^{H}$ and $m^{H}$, as follows:

(6) $${\hat{f}}^{H},{\hat{m}}^{H}=\underset{f^{H},m^{H}}{\arg\min}\left[-L(g|f^{H})+\beta R(f^{H},m^{H})\right],$$

where $L(g|f^{H})$ is the log-likelihood, *β* is the smoothing parameter that adjusts the balance between the likelihood term and the regularization term and $m^{H}$ is the median image of the underlying HR PET image $f^{H}$. As the derivation of the PL algorithm for SR reconstruction is essentially the same as that for non-SR reconstruction, for convenience, we drop the superscript H denoting “high resolution” for both **f** and **m** for the rest of the equations, so that our PL algorithm can be used for both SR and non-SR reconstructions.

To solve (6), its overall objective function is jointly minimized with respect to both f and m by using the following alternating algorithm:

(7) $$f^{n}=\underset{f}{\arg\min}\left[-L(g|f)+\beta R(f,m^{n-1})\right],$$

(8) $$m^{n}=\underset{m}{\arg\min}\left[R(f^{n},m)\right],$$

where *n* is the iteration number.

The early approach to minimizing the likelihood term in (7) was to use the expectation maximization (EM) algorithm [33]. Later, the EM algorithm was further improved to converge faster by employing block-iterative schemes. The popular ordered subsets EM (OSEM) algorithm [34] accelerates the convergent speed by subdividing the projection data into several subsets (or blocks) and then progressively processing each subset by performing projection and back-projection operations in each iteration. However, the OSEM algorithm does not have an objective function and converges to a limit cycle.

In this work, to overcome the limitation of the OSEM algorithm, we used a modified version of the COSEM algorithm [35], which is fast and convergent with an objective function. The COSEM algorithm applies the idea of ordered subsets used in the OSEM algorithm on the “complete data” **C** rather than on the projection data **g**. The complete data **C**, whose elements are denoted as $C_{ij}$, represent the number of coincidence events originated at a specific location in the underlying source and recorded by a specific detector pair, so that the following relationship holds: $\sum_{j}C_{ij}=g_{i}$. When the ML-based COSEM algorithm is extended to the PL approach, the overall objective function is modified to the following form:

(9) $$E\left(f;f^{n,l-1},C^{n,l},m^{n-1}\right)=-\sum_{ij}C_{ij}^{n,l}\log f_{j}+\sum_{ij}H_{ij}f_{j}+\beta R\left(f;f^{n,l-1},m^{n-1}\right).$$

In (9), *n* and *l* are the indices for the iteration and subset, respectively. For example, $f^{n,l}$ indicates the estimated image after processing the *l*-th subset of the *n*-th iteration. Once all of the subsets $\left\{S_{l}|l=1,\ldots ,L\right\}$ are successively processed, the *n*-th iteration is completed by setting $f^{n}=f^{n,L}.$

With the regularization term in (9), which takes the form of (3), it is not possible to obtain a closed-form solution. Therefore, we employed the method of optimization transfer using paraboloidal surrogates [36] that can efficiently find a global minimum of a convex function by using the following surrogate function for the regularization term $R\left(f;f^{n,l-1},m^{n-1}\right)$:

(10) $$U_{1}\left(f;f^{n,l-1},m^{n-1}\right)=\frac{1}{2}\sum_{j}\sum_{j^{\prime }\in N_{j}}w_{jj^{\prime }}\frac{\dot{\psi }\left(f_{j}^{n,l-1}-m_{j^{\prime }}^{n-1}\right)}{f_{j}^{n,l-1}-m_{j^{\prime }}^{n-1}}{\left(f_{j}-m_{j^{\prime }}^{n-1}\right)}^{2},$$

where $\dot{\psi }\left(\xi \right)$ is the first-order derivative of ψ(ξ). In (10), $w_{jj^{\prime }}$ is updated using (4), where the patch difference is calculated on $f^{n,l-1}$.

In this case, the overall surrogate objective function for (7) with respect to $f_{j}$ can be expressed as

(11) $$E_{s}\left(f_{j};f^{n,l-1},C^{n,l},m^{n-1}\right)=-\sum_{i}C_{ij}^{n,l}\log f_{j}+\sum_{i}H_{ij}f_{j}+\frac{\beta }{2}\sum_{j^{\prime }\in N_{j}}w_{jj^{\prime }}\frac{\dot{\psi }\left(f_{j}^{n,l-1}-m_{j^{\prime }}^{n-1}\right)}{f_{j}^{n,l-1}-m_{j^{\prime }}^{n-1}}{\left(f_{j}-m_{j^{\prime }}^{n-1}\right)}^{2}.$$

By taking the first-order derivative of (11) and setting it to zero, the following update equation for $f_{j}^{n,l}$ is obtained:

(12) $$f_{j}^{n,l}=\frac{-b+\sqrt{b^{2}-4ac}}{2a},$$

(13) $$a=\beta \sum_{j^{\prime }\in N_{j}}w_{jj^{\prime }}\frac{\dot{\psi }\left(f_{j}^{n,l-1}-m_{j^{\prime }}^{n-1}\right)}{f_{j}^{n,l-1}-m_{j^{\prime }}^{n-1}},$$

(14) $$b=\sum_{i}H_{ij}-\beta \sum_{j^{\prime }\in N_{j}}w_{jj^{\prime }}\frac{\dot{\psi }\left(f_{j}^{n,l-1}-m_{j^{\prime }}^{n-1}\right)}{f_{j}^{n,l-1}-m_{j^{\prime }}^{n-1}}m_{j^{\prime }}^{n-1},$$

(15) $$c=-\sum_{i}C_{ij}^{n,l}.$$

In (15), $C_{ij}^{n,l}{=g}_{i}\frac{H_{ij}f_{j}^{n,l-1}}{{\bar{g}}_{i}},\forall i\in S_{l}$ and $C_{ij}^{n,l}=C_{ij}^{n,l-1},\forall i\notin S_{l}$, where $S_{l}$ is the *l*-th subset. After all of the subsets $\left\{S_{l}|l=1,\ldots ,L\right\}$ are successively processed, the *n*-th iteration for **f** is completed by setting $f^{n}=f^{n,L}.$

The sub-minimization problem in (8) can be solved by minimizing the following surrogate objective function, which is similar to the surrogate function in (10) but uses *q* as the index for the sub-iteration:

(16) $$U_{2}\left(m;m^{n,q-1},f^{n}\right)=\frac{1}{2}\sum_{j}\sum_{j^{\prime }\in N_{j}}w_{j^{\prime }j}\frac{\dot{\psi }\left(f_{j^{\prime }}^{n}-m_{j}^{n,q-1}\right)}{f_{j^{\prime }}^{n}-m_{j}^{n,q-1}}{\left(m_{j}-f_{j^{\prime }}^{n}\right)}^{2},$$

where *q =* 1, …, *Q*. In (16), $w_{j^{\prime }j}$ is updated using (4), where the patch difference is calculated on $f^{n}.$

The surrogate objective function for (8) with respect to $m_{j}$ can be expressed as

(17) $$U_{3}\left(m_{j};m^{n,q-1},f^{n}\right)=\frac{1}{2}\sum_{j^{\prime }\in N_{j}}w_{j^{\prime }j}\frac{\dot{\psi }\left(f_{j^{\prime }}^{n}-m_{j}^{n,q-1}\right)}{f_{j^{\prime }}^{n}-m_{j}^{n,q-1}}{\left(m_{j}-f_{j^{\prime }}^{n}\right)}^{2}.$$

By taking the first-order derivative of (17) and setting it to zero, the following update equation for $m_{j}^{n,q}$ is obtained:

(18) $$m_{j}^{n,q}=\frac{\sum_{j^{\prime }\in N_{j}}w_{j^{\prime }j}\frac{\dot{\psi }\left(f_{j^{\prime }}^{n}-m_{j}^{n,q-1}\right)}{f_{j^{\prime }}^{n}-m_{j}^{n,q-1}}f_{j^{\prime }}^{n}}{\sum_{j^{\prime }\in N_{j}}w_{j^{\prime }j}\frac{\dot{\psi }\left(f_{j^{\prime }}^{n}-m_{j}^{n,q-1}\right)}{f_{j^{\prime }}^{n}-m_{j}^{n,q-1}}}$$

After a complete inner loop for the sub-iterations indexed by q=1,…,Q is finished, the *n*-th iteration for **m** is completed by setting $m^{n}=m^{n,Q}$.

The outline of the PL-SDMR algorithm is summarized in Algorithm 1, where f and m are updated alternately; for sub-minimization in (7), f is updated while fixing m, and, for sub-minimization in (8), m is updated while fixing f.
**Algorithm 1:** The outline of the PL-SDMR algorithm.Initialize f and m
            **for** each iteration *n* = 1,…,*N*              
$f^{n,0}=f^{n-1},$
              
$m^{n,0}=m^{n-1},$
               **for** each subset *l* = 1,…,*L*                  Update $f^{n,l}$ using (12),              
**end**
              
$f^{n}=f^{n,L},$
               **for** each sub-iteration *q* = 1,…,*Q*                  Update $m^{n,q}$ using (18),              
**end**
              
$m^{n}=m^{n,Q},$
        
**end**


## 3. Results

### 3.1. Reconstruction Accuracy

**Figure 2:**
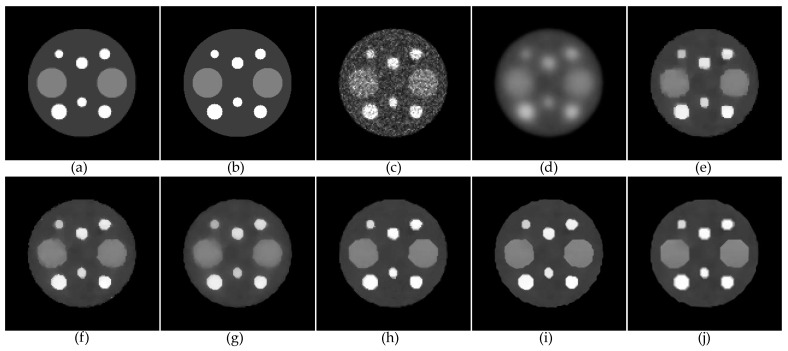
Anecdotal PL reconstructions: (**a**) LR phantom; (**b**) HR phantom; (**c**) LR-COSEM; (**d**) LR-LQR (MPE = 37.42%); (**e**) LR-LNQR (MPE = 19.27%); (**f**) LR-NWMR (MPE = 18.31%); (**g**) HR-NWMR (MPE = 18.89%); (**h**) LR-SDMR (MPE = 17.31%); (**i**) HR-SDMR (MPE = 15.77%); (**j**) HR-NLR (MPE = 16.85%).

To measure the reconstruction accuracy of our proposed method, we performed simulation studies using the mathematical phantoms of two different levels (LR with 128 × 128 pixels and HR with 256 × 256 pixels) of resolution. The LR phantom in Figure 2a was derived from the original HR phantom in Figure 2b by summing up four adjacent pixels to generate a corresponding pixel in the associated LR phantom. The projection data were generated from the LR phantom by our own projector with 128 bins and 128 discrete angles over 180 degrees. The gray scale of the phantom was adjusted to yield 500,000 projection counts. (The LR-COSEM result in Figure 2c qualitatively shows the Poisson noise level for the 500,000 projection counts.) Note that the HR phantom is only for measuring the error of HR reconstructions.

Here, we focused on the comparison of the SDMR with the NWMR in PL reconstruction. Both the SDMR and NWMR were applied to LR and HR (by SR) reconstructions to yield four variants of the NWMR, such as LR-SDMR, HR-SDMR, LR-NWMR and HR-NWMR (the HR images were reconstructed by our SR method). The number of subsets and the number of iterations used for reconstructing an image were 4 and 200, respectively, for all PL algorithms. To compare the SDMR with other existing regularizers, we additionally tested the PL algorithm with the local quadratic regularizer (LQR) and the local non-quadratic regularizer (LNQR) for LR reconstruction and the non-local regularizer (NLR) for HR reconstruction. The LQR takes a simple quadratic penalty, so that its first-order derivative, which represents the strength of smoothing, linearly increases as the intensity difference between the adjacent pixels increases. While the LQR suppresses noise well, it has an undesirable effect of oversmoothing edges. On the other hand, the LNQR takes a non-quadratic penalty, so that its first-order derivative does not increase further when the intensity difference between the adjacent pixels is relatively large at the edges. (Since both the LQR and LNQR do not perform as well as the NWMR/SDMR, only their LR reconstructions are shown in Figure 2. The reconstruction shown in Figure 2j is to give an idea of how the non-local patch-based quadratic regularization method, namely, the NLR method, compares with the SDMR in HR reconstruction.)

Figure 2d–j shows anecdotal reconstructions obtained with the seven different regularization methods described above. For the smoothing parameter *β*, we independently chose its value for each method so that the images reconstructed by the different regularization methods had approximately the same background noise level measured in the flat area of the largest circle. To measure the accuracy of each reconstruction, we calculated the mean percentage error (MPE) of each reconstruction from 50 independent Poisson noise trials, which is defined as

(19) $$\mathrm{MPE}=\frac{1}{K}\sum_{k=1}^{K}\sqrt{\sum_{j}{\left({\hat{f}}_{j}^{k}-f_{j}\right)}^{2}/\sum_{j}f_{j}{}^{2}}\times 100\%,$$

where ${\hat{f}}_{j}^{k}$ is the *j*-th pixel value of the reconstructed image for the *k*-th noise trial, $f_{j}$ is the *j*-th pixel value of the phantom and *K* = 50 is the total number of noise trials.

Figure 2d,e shows the LR reconstructions by the LQR and LNQR, respectively. Since the LQR cannot preserve edges, when the smoothing parameter was adjusted to yield the same background noise level as other edge-preserving regularizations, it resulted in a significantly oversmoothed reconstruction. For the LNQR, it clearly preserved edges while suppressing noise. Figure 2f,g shows the LR and HR reconstructions, respectively, by the NWMR. The visual comparison of Figure 2e with Figure 2f indicates that the NWMR reveals better recoveries of the circular shape with better contrast than the LNQR. The comparison of Figure 2f with Figure 2g indicates that, as the number of pixels per unit area increases from Figure 2f to Figure 2g, the image becomes less sharp, lowering the accuracy in terms of the MPE. When the NWMR was replaced with the SDMR, the accuracy of both LR and HR significantly increased by yielding sharper edges and better contrasts, which shows the efficacy of using the adaptive median weights determined by the patch similarities. (Compare the SDMR reconstructions in Figure 2h,i with the NWMR reconstructions in Figure 2f,g.) Then, one may expect that the HR-NLR would perform as well as the HR-SDMR. However, according to the results shown in Figure 2i,j, the HR-SDMR reconstruction in Figure 2i is sharper than the HR-NLR reconstruction in Figure 2j, which indicates that the NLR was not as effective in preserving sharp edges as the SDMR for this particular phantom with a relatively simple spatial structure.

**Figure 3:**
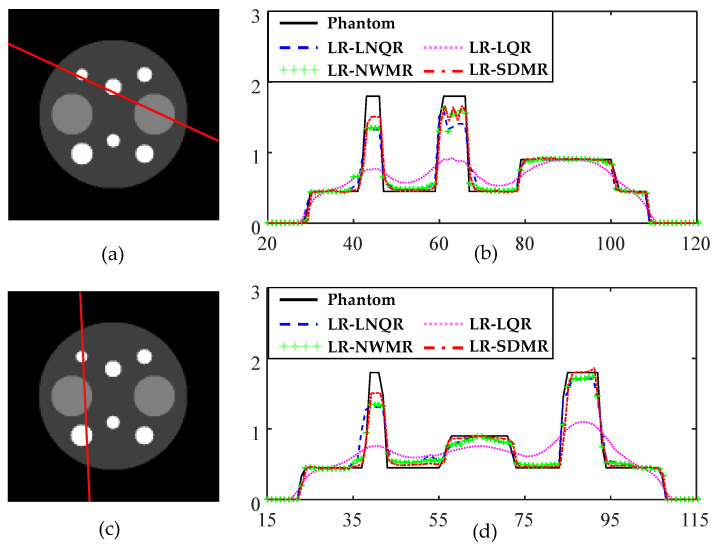
Profile plots for LR anecdotal reconstructions: (**a**) LR phantom with a profile line shown; (**b**) profile plots along the line shown in (**a**) for LR reconstruction methods (LNQR, LQR, NWMR and SDMR); (**c**) LR phantom with a profile line shown; (**d**) profile plots along the line shown in (**c**) for LR reconstruction methods (LNQR, LQR, NWMR and SDMR).

**Figure 4:**
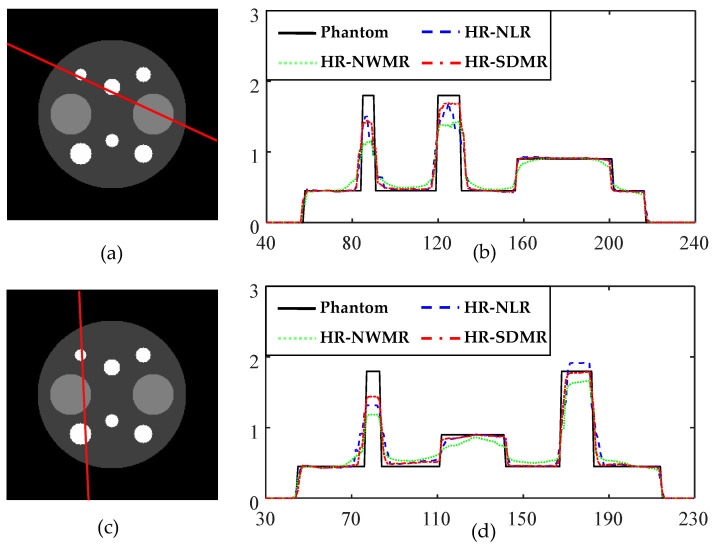
Profile plots for HR anecdotal reconstructions: (**a**) HR phantom with a profile line shown; (**b**) profile plots along the line shown in (**a**) for HR reconstruction methods (NLR, NWMR and SDMR); (**c**) HR phantom with a profile line shown; (**d**) profile plots along the line shown in (**c**) for HR reconstruction methods (NLR, NWMR and SDMR).

Figure 3a,c shows two lines on the LR image along which profiles are displayed for the four algorithms LR-LNQR, LR-LQR, LR-NWMR and LR-SDMR. Similarly, Figure 4a,c shows two lines on the HR image along which profiles are displayed for the three algorithms HR-NLR, HR-NWMR and HR-SDMR. (Note that the relative positions of the lines in Figure 4a,c are the same as those in Figure 3a,c, respectively.) From Figure 3b,d, it can be observed that the LR-SDMR not only incurred lower bias errors around pixels 40, 60 and 90 for both Figure 3b and Figure 3d, but also reconstructed most of the pixels more accurately than other regularizers used in the experiment.

Similar advantages of the SDMR can be observed from Figure 4b,d. (See around pixels 80, 130 and 180.) In addition, while the HR-NLR performed better than HR-NWMR, its overall performance was inferior to that of HR-SDMR.

**Figure 5:**
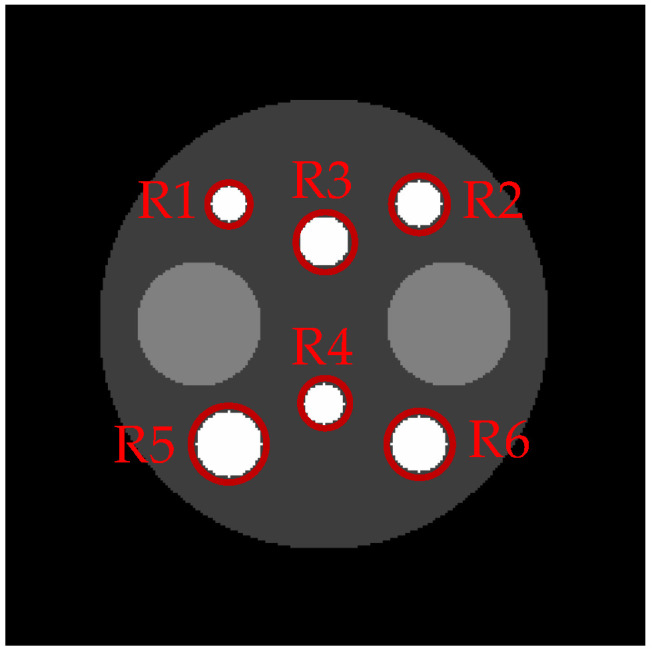
The six regions of interest for evaluating the regional performance of each algorithm used in Table 1.

To evaluate the regional performance of each algorithm, we first selected the six high-intensity circles (as shown in Figure 5) as regions of interest (ROIs) and computed the mean contrast recovery coefficients (MCRCs) of the reconstructions calculated from 50 independent noise trials. The regional MCRC defined in (20) measures how well the algorithm restores the contrast of an ROI with respect to its background chosen from the base circle with a low intensity.

(20) $$\bar{{\mathrm{CRC}}_{R}}=\sum_{k=1}^{K}{\mathrm{CRC}}_{R}^{k},\;\mathrm{with}\;{\mathrm{CRC}}_{R}={\mathrm{CR}}_{R}/{\mathrm{CR}}_{R0}.$$

In (20), $\bar{{\mathrm{CRC}}_{R}}$ stands for the regional MCRC, ${\mathrm{CR}}_{R}=\left|{\hat{A}}_{R}-{\hat{A}}_{Bg}\right|/{\hat{A}}_{Bg},$ where ${\hat{A}}_{R}=\left(1/T\right)\sum_{j\in R}{\hat{f}}_{j}$ denotes the mean activity for *T* pixels in each ROI, ${\hat{A}}_{Bg}$ is the mean activity in the background region and ${\mathrm{CR}}_{R0}$ is the true contrast in the phantom image.

Table 1 summarizes the regional MCRCs for the six ROIs, which indicates that the MCRC was significantly improved by using the SDMR-based methods (LR-SDMR and HR-SDMR). Note that the SDMR-based methods clearly outperformed the NWMR-based methods. In fact, the SDMR-based methods performed better than other methods except for R2, where the HR-NLR performed slightly better than the HR-SDMR, though the difference in the MCRC between the two methods was almost negligible. Since our SR method, to produce the HR reconstruction, involves upscaling, sometimes, the LR-SDMR revealed slightly better MRCRs than both the HR-SDMR and HR-NLR. (See the MCRCs for R3 and R4.)

To further validate the performance of our proposed method, we compared the accuracies of the reconstructed images shown in Figure 2 in terms of five different image quality assessment metrics, namely, mean structural similarity (MSSIM) [37,38], mean absolute error (MAE) [39], peak signal-to-noise ratio (PSNR) [37], root-mean-square error (RMSE) and visual information fidelity (VIF) [40]. As shown in Table 2, the proposed SDMR method performed better than the other non-SDMR methods tested here in all of the five metrics. In particular, the overall performance of the HR-SDMR method was clearly better than the other methods.

### 3.2. Robustness against Variation in the Smoothing Parameter

**Figure 6:**
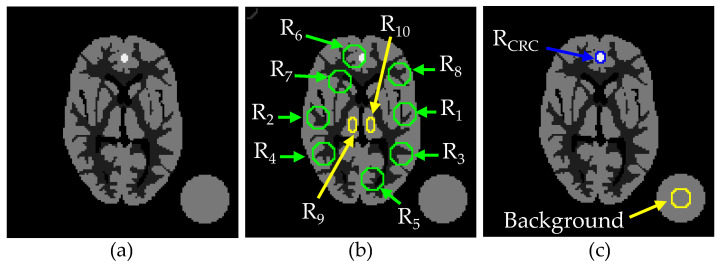
Hoffman brain phantom and ROIs for regional MPE and MCRC measures: (**a**) phantom; (**b**) ROIs for regional MPE; (**c**) ROI for CRC with respect to background.

To characterize how effective our method is while tested with different settings of the smoothing parameter β, we performed additional simulations using a new digital phantom, shown in Figure 6, where Figure 6a is a 128 × 128 Hoffman brain slice with a large circular area in the background to measure the noise level, Figure 6b shows ROIs for the quantitative regional studies and Figure 6c shows the ROI for the MCRC measure.

**Figure 7:**
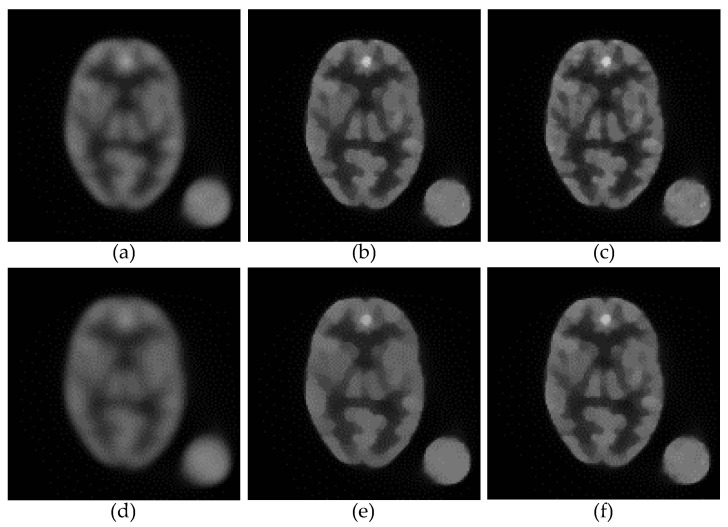
Anecdotal PL reconstructions: (**a**) LQR (*β* = 10, PE = 40.02%); (**b**) NWMR (*β* = 0.3, PE = 34.18%); (**c**) SDMR (*β* = 0.3, PE = 33.17%); (**d**) LQR (*β* = 25, PE = 43.95%); (**e**) NWMR (*β* = 0.6, PE = 36.37%); (**f**) SDMR (*β* = 0.6, PE = 34.47%).

Figure 7 shows the anecdotal reconstructions obtained from the PL algorithm with three different regularizers: Figure 7a,d, LQR; Figure 7b,e, NWMR; and Figure 7c,f, SDMR. To compare the performance of the different methods under the same conditions, we adjusted the smoothing parameter *β* of each method so that the resulting reconstructions had approximately the same background noise level measured in the circular area located outside of the phantom. Note that the background noise level of Figure 7b–d was higher than that of Figure 7e–g. When the smoothing parameters were adjusted to yield a relatively high background noise level, as shown in Figure 7b–d, the improvement, in terms of the percentage error (PE), from the NWMR to the SDMR was not stunning, though the SDMR reconstruction in Figure 7d was visually sharper than the NWMR reconstruction in Figure 7c. However, when the smoothing parameters were increased to yield a lower background noise level, as shown in Figure 7e–g, the SDMR clearly outperformed the NWMR in both qualitative and quantitative comparisons, which indicates that the SDMR not only outperformed the NWMR in reconstruction accuracy, but was also more robust to variations in the smoothing parameter.

**Figure 8:**
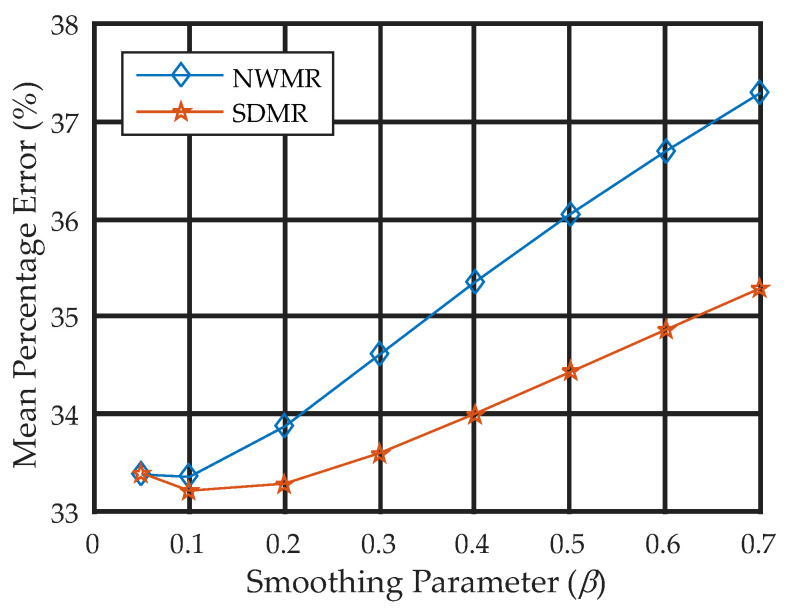
MPE-versus-smoothing parameter curves for NWMR and SDMR.

To observe the quantitative effect of changing the smoothing parameter *β*, we set the value of the smoothing parameter to the eight different values of *β* = 0.05, 0.1, 0.2, 0.3, 0.4, 0.5, 0.6 and 0.7 for each algorithm and measured the MPE over the 50 reconstructions obtained from 50 independent Poisson noise trials. Figure 8 shows the smoothing parameter-versus-MPE curves for the NWMR and SDMR methods. Note that, except for a small value of *β* = 0.05, the performance of the SDMR clearly outperformed the NWMR in terms of MPE over a range of the smoothing parameter.

To compare the results more quantitatively, we first pre-selected the 10 regions of interest (ROIs) as shown in Figure 6b and measured the regional MPEs over the 50 noise realizations of reconstructions for each method. Note that, while R_1_–R_8_ include sharp edges, R_9_ and R_10_ do not include an edge.

**Figure 9:**
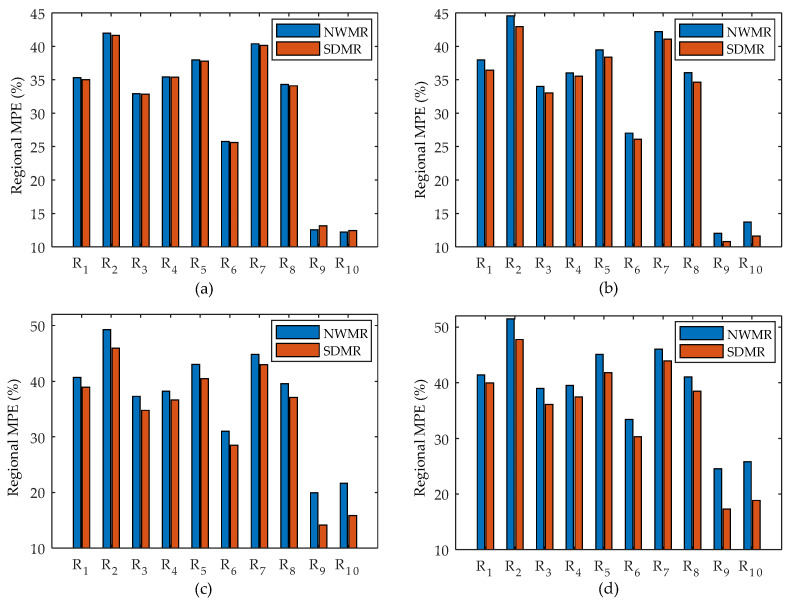
Error-bar plots for regional MPE for NWMR and SDMR: (**a**) *β* = 0.05; (**b**) *β* = 0.2; (**c**) *β* = 0.5; (**d**) *β* = 0.7.

Figure 9 shows the error-bar plots for the regional MPE of NWMR and SDMR, where the four samples (a) *β* = 0.05, (b) *β* = 0.2, (c) *β* = 0.4 and (d) *β* = 0.7 were chosen. According to the results in Figure 9, while the performance difference between the NWMR and SDMR was negligible for an extremely small value of *β*, which is almost equivalent to turning off the regularization term, it quickly became noticeable as soon as the smoothing parameter increased and was eventually significant for the large value of *β* = 0.7 in all regions. In particular, the SDMR significantly lowered the regional MPE in the small monotonic regions R_9_ and R_10_.

**Figure 10:**
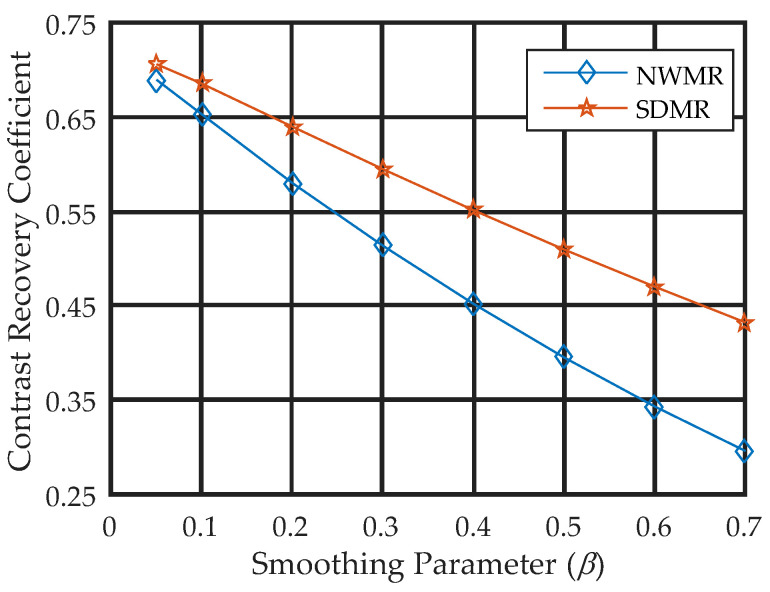
Regional MCRC curves evaluated over a range of the smoothing parameter *β* for NWMR and SDMR.

Figure 10 shows the regional MCRC curves over the range of β∈[0.05,0.7] calculated in the ROI with respect to the background designated in Figure 6c, where each regional MCRC was calculated from 50 independent noise trials. It is clear that the SDMR outperformed the NWMR over a wide range of the smoothing parameter. Moreover, the difference in the MCRC between the SDMR and NWMR became more significant as the smoothing parameter increased.

## 4. Summary and Conclusions

We introduce an adaptive method for selecting the weights for the median regularizer in penalized-likelihood PET reconstruction. In our method, the median weights are calculated in such a way that the higher the similarity between the center patch and its neighbor patch is, the more probable the median of the neighbor patch is to be chosen as the median of the center patch within a median window. In relatively smooth regions with occasional sharp edges, the SDMR performed just as the NWMR with a uniform weight. On the other hand, in regions containing many fine-scale edges, where most of the patches are not similar to each other, the weights of the SDMR became non-uniform, which made the SDMR adaptively preserve fine details more accurately than the NWMR.

We also applied the SDMR to the SR image reconstruction problem where the resolution of PET images increased during the iterative reconstruction process. As our SR method is applicable to any PL reconstruction methods involving repeated projection and back-projection operations, we also applied it to the PL reconstruction with the popular NLR and compared the resulting HR-NLR with the HR-SDMR. Our experimental results show that the SDMR provided more accurate HR reconstructions than the NLR in most of the image quality assessment metrics.

According to our additional test for a wide range of smoothing parameter settings, the SDMR was less sensitive to variations in the smoothing parameter than the NWMR. Moreover, for the same value of the smoothing parameter, the SDMR always outperformed the NWMR in terms of the MPE and MCRC. As the smoothing parameter increased, the difference in the MPEs, as well as that in the MCRCs, between the SDMR and NWMR became more significant.

In conclusion, while the NWMR has a fundamental limitation in preserving fine details, the proposed SDMR overcomes the limitation by adaptively selecting the median weights derived from the patch similarities that reflect the spatial structure of the underlying image. With this advantage, the SDMR has great potential for super-resolution reconstruction from low-resolution PET data without modification of scanner hardware. Finally, the SDMR is more robust to variations in the smoothing parameter than the NWMR, which indicates that the SDMR can be more reliable in practice. To validate the practical performance of the SDMR, further experiments with clinical data are required.

## Figures and Tables

**Table 1:** Regional MCRCs for ROIs in Figure 5 evaluated from 50 independent noise trials. The bold values indicate the best results.

ROI	LR-LQR	LR-LNQR	LR-NWMR	HR-NWMR	LR-SDMR	HR-SDMR	HR-NLR
R1	0.3131	0.7833	0.8046	0.7423	0.8777	**0.8805**	0.8791
R2	0.2199	0.7024	0.7165	0.6347	0.8090	0.8128	**0.8197**
R3	0.1986	0.6173	0.6303	0.5336	**0.7410**	0.7277	0.7384
R4	0.3148	0.8090	0.8164	0.7488	**0.8945**	0.8844	0.8896
R5	0.4454	0.8627	0.8740	0.8352	0.9256	**0.9317**	0.9205
R6	0.3853	0.8809	0.8886	0.8395	0.9482	**0.9543**	0.9302

**Table 2:** Quantitative performance comparison of reconstructions using five different image quality assessment metrics. The bold values indicate the best results.

Assessment Metrics	LR-LQR	LR-LNQR	LR-NWMR	HR-NWMR	LR-SDMR	HR-SDMR	HR-NLR
MSSIM	0.8647	0.9421	0.9467	0.9447	0.9525	**0.9593**	0.9550
MAE	5.1005	1.7672	1.6545	2.1027	1.2175	**1.1679**	1.3433
PSNR (dB)	20.6715	26.4239	26.8659	26.5827	27.3898	**28.1843**	27.6263
RMSE	0.0922	0.0475	0.0452	0.0467	0.0425	**0.0388**	0.0414
VIF	0.1340	0.2973	0.3224	0.3275	**0.3483**	0.3460	0.3394

## Data Availability

The data presented in this study are available upon request. Please contact the corresponding author.
